# To the Editors of the Medical and Physical Journal

**Published:** 1800-06

**Authors:** Thomas Peck

**Affiliations:** Higham Ferrars


*To the Editors of the Medical and Phyſical Journal.*

Gentlemen,

YOU will further oblige me by inſerting the following obſervations in your intereſting and eſteemed Journal. I am,

Gentlemen,

Your's, very reſpectfully,

THOMAS PECK.

*Higham Ferrars,*
*April 13, 1800.*

IN your laſt Journal, Mr. Davies very politely attempts an elucidation of his caſe of Adheſion of the Placenta, for which I thank him; and as he does not appear to be diſpleaſed with my commenting upon it in the *firſt*, I earneſtly hope he will not in a *ſecond* inſtance.

It is *indiſputably* allowed, that "a firm adheſion of a conſiderable part of the placenta to the internal ſurface of the uterus" conſtitutes an *obvious cauſe* of its retention, and when happening, *frequently* "formidable:" but, that the *hazard* of inverſion, or producing ſymptoms of irritation, (by perſevering in our attempts to extract it immediately) are *more* dangerous than the continuance of a conſiderable portion of it in utero, is ſtrongly ſuſpected. Mr. Davies, with great juſtneſs, remarks, that "Nature alone points out the propriety of an early delivery of the placenta by its *ſpeedy* expulſion after the birth of the fœtus." If Nature, then, (whoſe kind dictates we do well to imitate) ſo clearly demonſtrates this, it unqueſtionably behoves us to *aſſiſt* her when her efforts are *inſufficient.* An *early* contraction of the uterus after delivery, for evident reaſons, is earneſtly to be deſired; but, where the placenta (or a portion of it) attaches, *this* cannot take place. It therefore becomes our duty to ſeparate it as *quick as poſſible*; and if it does not adhere to the *fundus* uteri, very little difficulty will be experienced in its extraction; and even in *that* caſe, the trouble to the operator, or pain to the patient, will be *comparatively* trifling.

Mr. Davies, *in your laſt*, does not heſitate to *affirm*, that the *exhauſtion* of his patient was owing "to the *fatigue* occaſioned by the previous labour, together with the *irritation* produced in endeavouring to detach the placenta from its adheſion to the uterus; for no material hæmorrhage had taken place at *that time*." Yet, in *the account* of the caſe, we are told that Mr. Davies had loſt the common aid to the extraction of the placenta by the rupture of the funis. He then ſays, "*And* an hæmorrhage of *too conſiderable* a nature taking place to truſt it to to the natural efforts of the ſyſtem, I endeavoured to lay hold of the ſubſtance of it, and bring it away." Unfortunately, Mr. Davies did not effect this. He therefore, very properly, gave his patient ſome reſpite, and anticipated better ſucceſs at a little diſtance of time. "But," ſays he, "finding the *hæmorrhage rather alarming*, and the patient *ſinking*, I reſolved, in leſs than *an hour* from the firſt attempt, to make *another* effort, as the only alternative left to preſerve her life." The *hæmorrhage*, therefore, appears to have *given riſe* to Mr. Davies's fears for the ſafety of his patient—and the ſymptoms, faintings, colliquative ſweats, &c. moſt palpably aroſe from it: and though Mr. Davies appears to differ from me in attributing ſuch ſymptoms to exceſſive fatigue, I am ſtill of opinion they would not have exiſted but from the *hæmorrhage*.

I candidly admit the earneſt ſolicitude of Mr. Davies for the welfare of his patient, and ſincerely do I congratulate him on the happy termination of the caſe.

The grand object I have in view, is to prevent, as much as poſſible, a *too haſty* acquieſcence in that plan of treatment, which, *though ſucceſsful* in a *few* caſes, may probably conduce to laxity and ſupineneſs, and prove prejudicial in *others*; for, though the placenta *may* be retained for ſeveral days *with impunity*, yet ſhould the patient be loſt from any untoward circumſtance (in ſuch ſituation), the practitioner would incur cenſure for leaving *that* to nature *ſo long* which *timely* aſſiſtance might preclude the neceſſity of.

---

### *To the Editors of the Medical and Phyſical Journal.*

Gentlemen,

If you think the following caſes of Hydrocephalus Internus worthy a place in your valuable Journal, by inſerting them in your next Number you will much oblige,

Gentlemen,

